# Segmental Alterations of the Corpus Callosum in Progressive Supranuclear Palsy: A Multiparametric Magnetic Resonance Imaging Study

**DOI:** 10.3389/fnagi.2021.720634

**Published:** 2021-11-19

**Authors:** Lavinia A. Bârlescu, Hans-Peter Müller, Ingo Uttner, Albert C. Ludolph, Elmar H. Pinkhardt, Hans-Jürgen Huppertz, Jan Kassubek

**Affiliations:** ^1^Department of Neurology, University of Ulm, Ulm, Germany; ^2^German Center for Neurodegenerative Diseases (DZNE), Ulm, Germany; ^3^Swiss Epilepsy Clinic, Klinik Lengg, Zürich, Switzerland

**Keywords:** progressive supranuclear palsy, corpus callosum, magnetic resonance imaging, diffusion tensor imaging, texture analysis, fiber tracking, parkinsonian syndromes

## Abstract

**Background:** The regional distribution of the widespread cerebral morphological alterations in progressive supranuclear palsy (PSP) is considered to include segmental parts of the corpus callosum (CC).

**Objective:** The study was designed to investigate the regional white matter (WM) of the CC by T1 weighted magnetic resonance imaging (T1w MRI) data combined with diffusion tensor imaging (DTI) data in PSP patients, differentiated in the variants Richardson syndrome and PSP-parkinsonism, and to compare them with Parkinson’s Disease (PD) patients and healthy controls, in order to identify macro- and micro-structural alterations *in vivo*.

**Methods:** MRI-based WM mapping was used to perform an operator-independent segmentation for the different CC segments in 66 PSP patients vs. 66 PD patients vs. 44 matched healthy controls. The segmentation was followed by both planimetric and texture analysis of the separated CC areas for the comparison of the three groups. Results were complemented by a DTI-based tract-of-interest analysis of the associated callosal tracts.

**Results:** Significant alterations of the parameters *entropy* and *homogeneity* compared to controls were observed for PSP as well as for PD for the CC areas I, II, and III. The inhomogeneity in area II in the PSP cohort was the highest and differed significantly from PD. A combined score was defined as a potential marker for the different types of neurodegenerative parkinsonism; receiver operating characteristics (ROC) curves were calculated with areas under the curve values of 0.86 for PSP vs. controls, 0.72 for PD vs. controls, and 0.69 for PSP vs. PD, respectively.

**Conclusion:** The multiparametric MRI texture and DTI analysis demonstrated extensive alterations of the frontal CC in neurodegenerative parkinsonism, whereas regional CC atrophy cannot be regarded as a constant neuroimaging feature of PSP. Specifically, the comparison PSP vs. PD revealed significant alterations in callosal area II. The combination of the texture and the DTI parameters might contribute as a neuroimaging marker for the assessment of the CC in PSP, including the differentiation vs. PD.

## Introduction

Progressive supranuclear palsy (PSP) as one cause of atypical neurodegenerative parkinsonism is now recognized as a range of movement, behavioral, and language syndromes associated with a characteristic 4-repeat tau neuropathology (Boxer et al., 2017), pathologically characterized by tau protein deposition, neuronal loss, and gliosis affecting the brainstem, subcortical, and cortical structures (Litvan et al., 1996; Kovacs et al., 2020). As mirrored in the MDS PSP Diagnostic Criteria (Höglinger et al., 2017; Grimm et al., 2019), different clinical subtypes have been described beyond the “classical” presentation as Richardson’s syndrome (PSP-RS), including most commonly the subtype characterized by a more Parkinson’s disease (PD)-like presentation, i.e., PSP-parkinsonism (PSP-P), but also various additional clinical phenotypes (Respondek et al., 2017). In the diagnostic workup, magnetic resonance imaging (MRI) signs of PSP primarily include prominent midbrain and frontal atrophy (Whitwell et al., 2017). Currently, the automatically calculated MR parkinsonism index, comprised of pons, midbrain, and peduncular measures, in its new version including the measurement of the third ventricle width (Quattrone et al., 2018), has successfully been used for multisite MRI data (Nigro et al., 2020). Beyond structural MRI approaches, various MRI techniques have previously been applied to PSP to demonstrate abnormalities in frontoparietal/frontotemporo-occipital connections (Agosta et al., 2012; Whitwell et al., 2017; Kassubek, 2018). Diffusion-weighted MRI (in combination with machine learning approaches, e.g., automated imaging differentiation) has been shown to be capable of differentiating forms of parkinsonism (Archer et al., 2019). Recent MRI approaches to PSP by diffusion weighted imaging focused on the differentiation of the findings between the different phenotypic variants of PSP (Potrusil et al., 2020; Whitwell et al., 2021). However, the corpus callosum (CC) has not been an anatomical area of specific interest in this context yet and has been addressed only in limited numbers of patients by computerized MRI sensitive to microstructural changes (Rosskopf et al., 2014) and by surface-based analysis (Lenka et al., 2017), although also routine structural MRI in PSP might demonstrate callosal atrophy at individual level, especially in its frontal areas. A recent innovative whole brain-based study applying fixel-based analysis to diffusion weighted imaging in patients with neurodegenerative parkinsonism (including PSP and PD) demonstrated specific patterns of white matter degeneration which included, in PSP, the body of the CC together with the superior corona radiata, internal capsules, and the midbrain (Nguyen et al., 2021).

The CC is increasingly recognized as an important structure to demonstrate regional WM degeneration in different neurodegenerative diseases. In motor neuron diseases, a disease spectrum characterized by a prominent corticospinal tract degeneration, a focal lesion of the motor area III according to the scheme proposed by Hofer and Frahm (2006) could be demonstrated (Müller et al., 2020), and the same segment had been described to be microstructurally altered in corticobasal degeneration by diffusion tensor imaging (Boelmans et al., 2006).

Based on the above-named results of previous neuroimaging studies in PSP, we intended to investigate the CC as the region-of-interest in order to identify specific PSP-associated alterations. To this end, the involvement of the CC in PSP was investigated with the aim to identify a potential multiparametric MRI marker by objective parametrization: in addition to texture analysis, the fiber structures of the CC were investigated. A new parameter combining texture analysis of the (planar) CC and the callosal fiber structures was defined. The aim of the study was first, to compare *in vivo* the regional findings of the CC in PSP with those in PD and second, to investigate potential differences between the variants PSP-RS and PSP-P, with respect to macro- and micro-structural alterations.

## Materials and Methods

### Subjects and Patient Characteristics

Patients were selected who were diagnosed with PSP between 2005 and 2019 in the Department of Neurology, University of Ulm, Germany with in-house cranial MRI data available within 6 months prior to or after the time of diagnosis. Only patients with available documented clinical data sufficient to review the PSP diagnosis with the current diagnostic criteria established by the Movement Disorder Society (MDS)–endorsed PSP Study Group (MDS criteria, Höglinger et al., 2017), and patients who met these new criteria were selected. Also, patients were excluded if the cranial MRI did not contain 3-D T1w and DTI data or had limited acquisition quality (e.g., motion artifacts). Only the clinical subtypes PSP-RS and PSP-P were included for the analysis resulting in a total of 46 PSP-RS patients and 20 PSP-P patients, regardless of the diagnostic certainty level.

These 66 PSP patients underwent standardized clinical and neurological examinations focusing on movement and cognitive abnormalities. Clinical diagnoses for all patients were established by at least one trained neurologist with more than 10 years of experience in movement disorders using international diagnostic criteria for PSP and PD, respectively (Hughes et al., 1992; Litvan et al., 1996; Gelb et al., 1999; Höglinger et al., 2017). PSP patients enrolled before 2017 were reclassified according to the recent clinical diagnostic criteria for PSP-RS and PSP-P (Höglinger et al., 2017, Grimm et al., 2019). All PSP patients were categorized in predominant phenotypes (PSP-RS and PSP-P) and levels of diagnostic certainty (probable, possible, and suggestive of PSP) according to the MDS diagnostic criteria for PSP. In detail, the eponymous vertical ocular motor dysfunction was stratified by two levels of certainty according the new PSP diagnostic criteria: O1 (highest level) and O2 (mid-level, slowness of vertical saccades) (Wunderlich et al., 2021). The O1 and O2 levels had to be associated with postural instability (P1, repeated unprovoked falls within 3 years; or P2, tendency to fall on the pull test within 3 years) for a diagnosis of PSP-RS, while these ocular signs had to be associated with A2 (levodopa-resistant parkinsonism) or A3 (levodopa-responsive parkinsonism) for a diagnosis of PSP-P. Both groups PSP-RS and PSP-P contained patients with the diagnostic certainty levels probable and possible (Table 1). The exclusion criteria for patients were: other major systemic, psychiatric or neurological illnesses, history of neuroleptic use within the past 6 months, any brain or cervical cord abnormalities visible on routine MRI scans and suggesting a different etiology of the clinical symptoms, in particular evidence of vascular lesions such as lacunar infarctions in the basal ganglia and/or subcortical vascular lesions with diffuse periventricular signal alterations or radiologic signs suggestive of normal pressure hydrocephalus.

**TABLE 1:** Subjects’ characteristics.

	PSP (all)	PSP-RS	PSP-P	PD	Controls	*p*
Male/female	38/28	24/22	14/6	41/25	25/19	Kruskal-Wallis:0.7
Age/years (mean ± std. dev.)	71 ± 9 (49– 91)	71 ± 9 (49– 84)	70 ± 9 (50– 91)	71 ± 10 (52 –94)	69 ± 5 (61 –81)	Kruskal-Wallis:0.3
Disease duration/years (mean ± std. dev.)	3.1 ± 1.8	2.7 ± 1.5	4.0 ± 2.2	3.6 ± 2.6	–	*t*-test: 0.15
Hoehn and Yahr (mean ± std. dev.)	3.4 ± 0.7	3.3 ± 0.7	3.5 ± 0.8	2.6 ± 1.0	–	*t*-test: < 0.01

*p, PSP vs. PD vs. controls.*

The PSP patients’ data were compared with were compared with a disease control group of 66 age- and gender-matched subjects with Parkinson’s disease (PD). All PD patients fulfilled strict diagnostic criteria for idiopathic PD according to UK Parkinson’s Disease Society Brain Bank criteria (Hughes et al., 1992). In addition, a group of 44 age- and gender-matched healthy controls was investigated. All subjects gave written informed consent for the study protocol according to institutional guidelines which had been approved by the Ethics Committee of Ulm University, Germany (reference # 279/19).

### Magnetic Resonance Imaging Acquisition

Magnetic resonance imaging scanning was performed on a 1.5 Tesla Magnetom Symphony (Siemens Medical, Erlangen, Germany); the study protocol consisted of a T1w scan with 144 slices [256 × 256 pixels, slice thickness 1.2 mm, pixel size 1.0 mm x 1.0 mm, echo time (TE) 4.2 ms, repetition time (TR) 1640 ms] and a diffusion tensor imaging (DTI) study protocol with 52 volumes (64 slices, 128 × 128 pixels, slice thickness 2.8 mm, pixel size 2.0 mm x 2.0 mm), 48 gradient directions (*b* = 1000 s/mm^2^), and four scans with *b* = 0, TE 95 ms, TR 8,000 ms.

### Multiparametric Magnetic Resonance Imaging Data Analysis

The pre- and postprocessing were performed by use of the analysis software *Tensor Imaging and Fiber Tracking* (*TIFT*; Müller et al., 2007).

#### Texture Analysis

Details of the analysis cascade have been described previously (Müller et al., 2020; Müller et al., 2021). In short, after isometric and affine alignment to the anterior commissure/posterior commissure line and adjustment of the intensity threshold to automatically segment the CC, a subdivision of the CC into areas I–V according to the Hofer and Frahm scheme (Hofer and Frahm, 2006) was performed. Finally, calculation of area sizes and texture parameters (Stockman and Shapiro, 2001) was applied.

In the current study, the parameters entropy and homogeneity were analyzed; the entropy in a given sample increases when the distribution of gray values in the sample shows a more inhomogeneous pattern, while the parameter homogeneity rises when gray level differences between neighboring voxels increase (which means that in fact, the structural inhomogeneity rises). Therefore, in the following, we will use the term inhomogeneity for alterations of the tissue property described by the parameter homogeneity. That way, texture parameters could be candidates to identify focal microstructural alterations which are not seen as atrophy. Entropy and homogeneity were chosen as candidate parameters, since these parameters had performed best in a previous study on discrimination of callosal texture in neurodegenerative motor neuron disorders and controls (Müller et al., 2020). Therefore, further first- and second-order features (like skewness, kurtosis, correlation, and energy) were not analyzed in the current study.

#### Corpus Callosum Segmentation and Planimetry by Atlas-Based Volumetry

As a reference analysis for segmentation and planimetry of the CC, the standardized unbiased approach of Atlas-Based Volumetry (ABV) (Huppertz et al., 2010, 2016) was used. For the purpose of the current study and as described in detail elsewhere (Rosskopf et al., 2014), the area sizes of the midsagittal CC plane and its segments I–V according to the definition by Hofer and Frahm (2006) were measured.

#### Microstructure Analysis

In order to identify microstructural alterations in 3-D tracts originating in the five segmented callosal areas, DTI data were analyzed. The DTI analysis algorithms have been previously described in detail (e.g., Müller et al., 2009; Kassubek et al., 2018). In short, stereotaxic normalization to the Montreal Neurological Institute (MNI) space was iteratively performed using study-specific templates (Müller et al., 2009), and FA maps were quantitatively calculated from the stereotaxically normalized DTI data sets of all subjects. Prior to correction for the covariate age, a Gaussian filter of 8 mm full width at half maximum was applied for smoothing of FA maps (Unrath et al., 2010). From the MNI normalized data of 44 controls, an averaged data set was calculated; this data set was used for fiber tracking. To this end, a conventional streamline tracking was used with a vector product threshold of 0.9 and an FA threshold of 0.2. The CC could be subdivided into five areas (Hofer and Frahm, 2006): area I are callosal fibers comprising bundles projecting into the prefrontal lobe, area II are callosal fibers projecting to frontal areas including premotor and supplementary motor cortices, area III fibers project to the primary motor cortices, area IV fibers project to primary sensory cortices, and area V fibers project to parietal lobe, occipital lobe, and temporal lobe. Defined tracts in areas I to V of the CC according to this scheme were identified with the TOI approach (Kassubek et al., 2018). Tractwise fractional anisotropy statistics (TFAS) (Müller et al., 2009) was performed by statistically comparing the FA values in each tract between the two groups (Student’s *t*-test), not considering FA-values below 0.2, since cortical gray matter shows FA values up to 0.2 (Kunimatsu et al., 2004).

#### Combined Score

Z-normalization was performed for FA and texture (entropy and homogeneity) results, and the parameters were summarized to a single combined score C with a weighting of 1: 0.5: 0.5, i. e.,


(1) $$C=\left({\text{z}}_{F\,A}+0.5\,{\text{z}}_{e\,n\,t\,r\,o\,p\,y}+0.5\,{\text{z}}_{h\,o\,m\,o\,g\,e\,n\,e\,i\,t\,y}\right)/2.$$

The rationale of the weighting is an equal weighting between microstructure (represented by FA with a weighting factor of 1) and texture (represented by the two parameters entropy and homogeneity so that each received a factor of 0.5).

#### Statistical Comparison at the Group Level

The subject groups showed a Gaussian distribution of parameters, and Student’s *t*-test was used for comparison at the group level. All the results for the comparisons PSP vs. controls, PD vs. controls, and PSP vs. PD for the five callosal areas, respectively, were corrected for multiple comparisons (Bonferroni-corrected). To provide a measure of the discriminative power of the combined score, receiver operating characteristics (ROC) curves were calculated.

## Results

### Subjects‘ Demographic and Cognitive Data

The subjects with PSP (38m/28f, age 71 ± 9 years, mean disease duration 3.1 ± 1.8 years) were compared 66 subjects with PD (41m/25f, age 71 ± 10 years, mean disease duration 3.6 ± 2.6 years) the 44 age- and gender-matched healthy controls (25m/19f, age 69 ± 5 years) – for detailed demographic and clinical data of these two groups, see Table 1.

Out of the 66 PSP patients, 31 underwent the complete CERAD-plus-battery, while 19 patients were assessed with parts of this test. For the remaining 16 PSP patients, the cognitive status was clinically assessed by an unstructured interview. Due to the inhomogeneity in the neuropsychological assessment, the cognitive status was dichotomized, i.e., the patients were characterized as neuropsychologically impaired (*N* = 47) if they met the criteria for frontal cognitive/behavioral presentation (according to the MDS PSP criteria (Höglinger et al., 2017). If they did not meet the criteria, the patients were characterized as unimpaired/normal (*N* = 19).

### Plane Size of the Areas I – V of the Corpus Callosum

**FIGURE 1:**
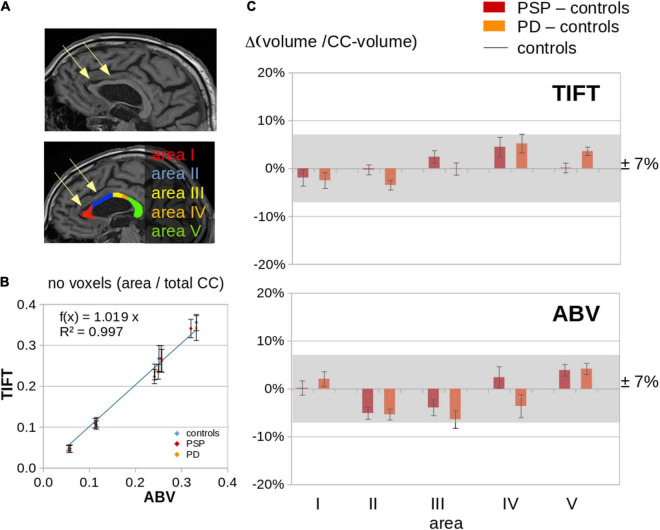
ABV and planimetry (TIFT) for the subject groups. **(A)** Examples of a PSP patients with visually apparent regional volume alterations in callosal areas I and II and the respective CC segmentation. **(B)** Association of ABV and planimetry (TIFT) results for subject groups controls, PSP, and PD, separated into callosal areas; number of voxels were provided as ratio of number of voxels in the respective callosal area and the total number of voxels in the CC. **(C)** Comparison of ABV and planimetry (TIFT) results of PSP and PD patients and controls; “volume/CC-volume” means the ratio of number of voxels in the respective callosal area and the total number of voxels in the CC. Results of PSP patients and PD patients were normalized to the average callosal area of controls (“PSP – controls” and “PD – controls”).

In the T1w images of the PSP patients, apparent atrophy in the frontal CC could sometimes be detected by visual inspection in individual patients (for examples, see Figure 1A). To detect whether this is a common feature in PSP and/or PD, this imaging sign was investigated by planimetry of the midsagittal CC areas, using ABV and TIFT for mutual comparison and validation. Methodologically, the results for ABV-based and TIFT-based planimetry showed a high correlation at the group level (*R*^2^ = 0.997) (Figure 1B).

In patients with PSP and patients with PD, respectively, no significant alterations of CC areas I – V could be detected at the group level when compared to controls, not even in callosal areas I and II which sometimes appeared visually reduced at single case level (Figure 1C). In summary, these results indicated that focal atrophy of the CC is not a neuroimaging feature of PSP or PD.

### Tract-Based DTI Results

**FIGURE 2:**
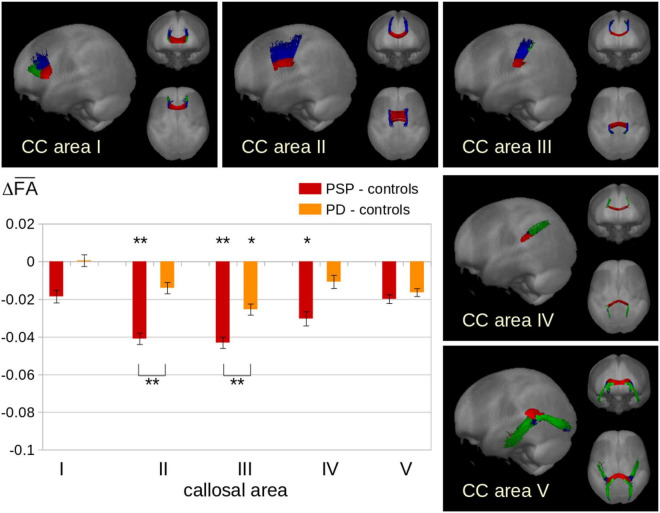
Tractwise fractional anisotropy statistics (TFAS) for fiber tracts originating in callosal areas. Displayed are the differences (Δ) in average FA values between patient groups and controls. PSP showed highly significantly reduced FA at the group level especially in areas II and III, while PD showed significantly reduced FA (however at a lower level compared to PSP) only in area III. *p < 0.01, **p < 0.001, corrected for multiple comparisons.

Tractwise fractional anisotropy statistics for PSP showed highly significant alterations in CC areas I, II, and III compared to controls and also compared to PD (Figure 2), whereas for PD, significant alterations compared to controls could be detected only in area III.

### Alterations of Texture of the Corpus Callosum

**FIGURE 3:**
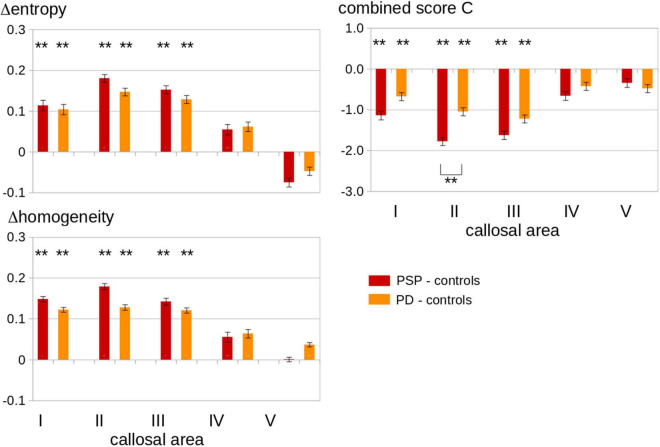
**Left Panel:** Texture results for CC areas I–V in the patients with PSP as compared to patients with PD and to healthy controls. Displayed are the differences (Δ) in average values of the parameters entropy and homogeneity between the two patient groups and the controls. PSP patients showed an increased entropy and homogeneity of CC area I, area II, and area III compared to controls. Entropy and homogeneity of CC area I, area II, and area III were increased in PD compared to controls. **Right panel:** Combined score C (see Eq. 1) results for CC areas I-V in the patients with PSP as compared to the patients with PD and to healthy controls. Displayed are the differences (Δ) in average C values between patient groups and controls. PSP patients and PD patients showed increased C values in callosal areas I, II, and III compared to controls, while areas IV and V showed no significant differences. Significant differences between PSP patients and PD patients were shown by brackets below the column (only for callosal area II). Error bars are the standard error of the mean. **p < 0.001, corrected for multiple comparisons.

The texture parameters for patients with PSP, patients with PD, and healthy controls at the group level are summarized in Figure 3. The results for patient groups are presented as the difference to the mean values of controls. PSP patients and PD patients showed increased entropy and increased inhomogeneity in the texture of CC areas I, II, and III compared to controls, while areas IV and V showed no significant differences. No significant association was found for disease duration.

The texture parameters for patients with PSP-RS and PSP-P and patients with PD at the group level are summarized in Supplementary Figure 1 (the results for the patient groups are presented as differences to the mean values of controls). PSP-RS patients and PSP-P patients showed increased entropy and increased inhomogeneity in the texture of CC areas I, II, and III compared to controls, while areas IV and V showed no significant differences. No significant difference was observed between PSP-RS patients and PSP-P patients. For the combined parameter C, the separation into PSP-RS and PSP-P showed more marked alterations for PSP-RS than for PSP-P; in areas II and III, these alterations were significant for PSP-RS compared to PD patients, whereas the alterations of PSP-P were not different from the findings in PD.

### Combined Score

The combined scores for patients with PSP, patients with PD, and healthy controls at the group level are summarized in Figure 3. The parameter C combines texture alterations in the central sagittal slice in the CC with microstructural alterations in 3-D tracts originating in callosal areas, that way increasing the signal-to-noise ratio. PSP patients and PD patients showed reduced C scores in callosal areas I, II, and III compared to controls, while no significant differences were found for CC areas IV and V. These significant differences to controls were also observed for each parameter FA, entropy, and homogeneity alone, but the C-score showed highly significant alterations only for area II when comparing PSP patients and PD patients, in contrast to FA analysis alone which yielded significant alterations both in areas II and III.

### Receiver Operating Characteristics for the Combined Score C of Callosal Area II

**FIGURE 4:**
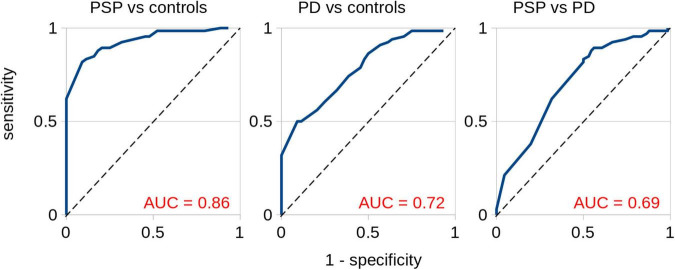
Receiver operating characteristics for the combined score C of callosal area II. Separation for PSP vs. controls, PD vs. controls, and PSP vs. PD. The areas under the curve (AUC) values were 0.86 (excellent) for PSP vs. controls, 0.72 (acceptable) for PD vs. controls, and 0.69 (hardly acceptable) for PSP vs. PD.

In order to provide a measure of the discriminative power of the combined score of callosal area II as a potential marker for different types of neurodegenerative parkinsonism, receiver operating characteristics (ROC) curves were calculated. The areas under the curve (AUC) values were 0.86 (excellent) for controls vs. PSP, 0.72 (acceptable) for controls vs. PD, and 0.69 for PSP vs. PD (hardly acceptable), respectively (Figure 4).

## Discussion

This multiparametric MRI study which combined a T1w MRI texture analysis of the CC segments and a DTI-based tract-of-interest analysis of the corresponding callosal tracts demonstrated that the parameters entropy and homogeneity were regionally altered in CC areas I, II, and III both in PSP and in PD as compared to controls, thus constituting a common feature of both types of neurodegenerative parkinsonism. In the combination of texture analysis and tract-based analysis, the highest alterations were observed in the CC area II which can be regarded as an *in vivo* estimate of PSP-associated structural brain alterations in frontal (premotor) areas. The DTI approach specifically addressed the callosal fibers which fan out into the white matter of both hemispheres, i.e., the radiation of the CC; here, the homotopic commissural connections which interconnect the frontal lobes pass in the anterior half of the CC (Nieuwenhuys et al., 2019). By means of the current results, the DTI-based findings of a pilot study (Rosskopf et al., 2014), i.e., frontal CC alterations in PSP but not in PD, could be validated. The current findings demonstrated that the maximum of alterations focused on CC area II (as confirmed by the combined parameter C). Given that the location of the atrophy within the CC is related to the location of cortical atrophy, the predominance of atrophy in the anterior CC portion is in line with the main focus of PSP-associated cortical atrophy being localized in the medial frontal lobes (Boxer et al., 2017). On the other hand, although individual PSP patients can show marked deformations and/or atrophy of the frontal CC in morphological images (personal observation; for example, see Figure 1A), this finding was not confirmed by planimetry at the group level in the current study and cannot be regarded as a constant neuroimaging feature of PSP. The investigation of the size of CC areas showed no differences between PSP patients, PD patients, and controls, i.e., the quantification algorithm was apparently not sensitive to a finding that probably is more a deformation than an atrophy.

For a more subtle T1w MRI analysis, thus, the entropy and the homogeneity were also analyzed in the current study. The entropy in a given sample increases when the gray value distribution shows a more inhomogeneous pattern, while structural inhomogeneity rises when gray level differences between neighboring voxels increase. PSP was associated with significant regional alterations of entropy and homogeneity in areas I - III of the CC. When compared with PD, however, there were no significant differences between the two groups, since PD was also associated with frontal CC alterations compared to controls. This involvement of the CC in the pathological process of PD supports previous neuroimaging studies which reported wide structural disruptions in the anterior callosal subsections (Bledsoe et al., 2018) and showed decreased FA and increased mean diffusivity in the whole CC and its subsections except the temporal-parietal-occipital subsection (Wu et al., 2020). The findings in PSP, however, were beyond these PD-associated alterations, since in PSP patients, the introduction of a combined parameter score led to a comprehensive textural/microstructural data analysis which showed alterations in frontal CC areas, most prominent in the callosal area II which were significant in comparison to PD patients. The more prominent CC alterations in PSP are in a line of agreement with the findings of a pilot study employing binary pattern-based texture analysis in both disorders which described more localized tissue alterations in mid-callosal regions in PSP (Bhattacharya et al., 2020). Our findings are also in a line of agreement with a fixel-based study in patients with neurodegenerative parkinsonism which demonstrated a reduction of a combined measure of fiber density and cross-section in the body of the CC in PSP (Nguyen et al., 2021). As the pathological basis for the focal alterations of the frontal corpus callosum, a correlate of the frontal lobe involvement might be suggested, as demonstrated by the distribution pattern of tau pathology in PSP (Kovacs et al., 2020). The potential direct functional correlates of the involvement of the premotor subsection of the CC cannot be unequivocally differentiated yet within the clinical syndrome of PSP. A correlation with the PSP-associated frontal dysexecutive impairments might be addressed in future studies combining neuroimaging and detailed neuropsychological assessment. For the combined score, the PSP subtype PSP-RS showed significantly altered values focused in the callosal areas II and III, compared both with controls and with PD, whereas the subtype PSP-P showed similar scores as PD, i.e., the significant alterations in PSP were essentially guided by the PSP-RS group, while PSP-P alterations resembled those found between PD and controls. These results add to the findings of a previous automated surface-based analysis in which the PSP-RS group had lower CC area III size compared to the PSP-P group (Lenka et al., 2017).

The study was not without limitations. The current MRI data demonstrated PSP-associated CC alterations at group level, but provide no marker that contributes to an accurate clinical diagnosis with sufficient sensitivity and specificity at single patient level so far. The design was cross-sectional, and thus results will have to be re-evaluated in a longitudinal design in future studies. In addition, neuropathological confirmation of the diagnosis by autopsy results was not available. Furthermore, a detailed standardized neuropsychological assessment is needed to analyze the role of the texture differences of the CC with respect to the cognitive clinical PSP phenotypes.

In summary, the combined MRI score encompassing FA, entropy, and homogeneity for the CC might be included as a candidate for a neuroimaging marker in PSP. Specifically for the CC segment II, the current ROC analyses indicate a potential to discriminate between PSP and PD. This multiparametric MRI analysis of the CC demonstrated a specific involvement of its frontal subsections in PSP-RS, in accordance with the frontal involvement as step 4 in the proposed staging scheme for the neuropathological practice in PSP with increased neuronal tau pathology in the frontal lobe (Kovacs et al., 2020), while the CC itself has not been specifically addressed in the underlying neuropathological study. These findings will have to be challenged in future studies with longitudinal data and with patients with neurodegenerative parkinsonism in earlier clinical disease stages.

## Data Availability Statement

The original contributions presented in the study are included in the article/Supplementary Material, further inquiries can be directed to the corresponding author.

## Ethics Statement

The studies involving human participants were reviewed and approved by Ethics Committee of Ulm University, Germany. Written informed consent for participation was not required for this study in accordance with the National Legislation and the Institutional Requirements.

## Author Contributions

LB: data collection, data analysis, and drafting of manuscript. H-PM: study concept and design, data analysis, interpretation of data, and drafting of manuscript. IU, AL, and EP: interpretation of data and critical revision of manuscript for intellectual content. H-JH: data analysis and interpretation of data, and critical revision of manuscript for intellectual content. JK: study concept and design, interpretation of data, study supervision, and drafting of manuscript. All authors contributed to the article and approved the submitted version.

## Conflict of Interest

The authors declare that the research was conducted in the absence of any commercial or financial relationships that could be construed as a potential conflict of interest.

## Publisher’s Note

All claims expressed in this article are solely those of the authors and do not necessarily represent those of their affiliated organizations, or those of the publisher, the editors and the reviewers. Any product that may be evaluated in this article, or claim that may be made by its manufacturer, is not guaranteed or endorsed by the publisher.
