# Supplementary material for: Segmental Alterations of the Corpus Callosum in Progressive Supranuclear Palsy: A Multiparametric Magnetic Resonance Imaging Study
Source: Front Aging Neurosci. 2021 Nov 19;13:720634. doi: 10.3389/fnagi.2021.720634 (PMC8640496; doi:10.3389/fnagi.2021.720634)
Supplement: Supplementary file 1 [file Data_Sheet_1.docx]

Supplementary Material


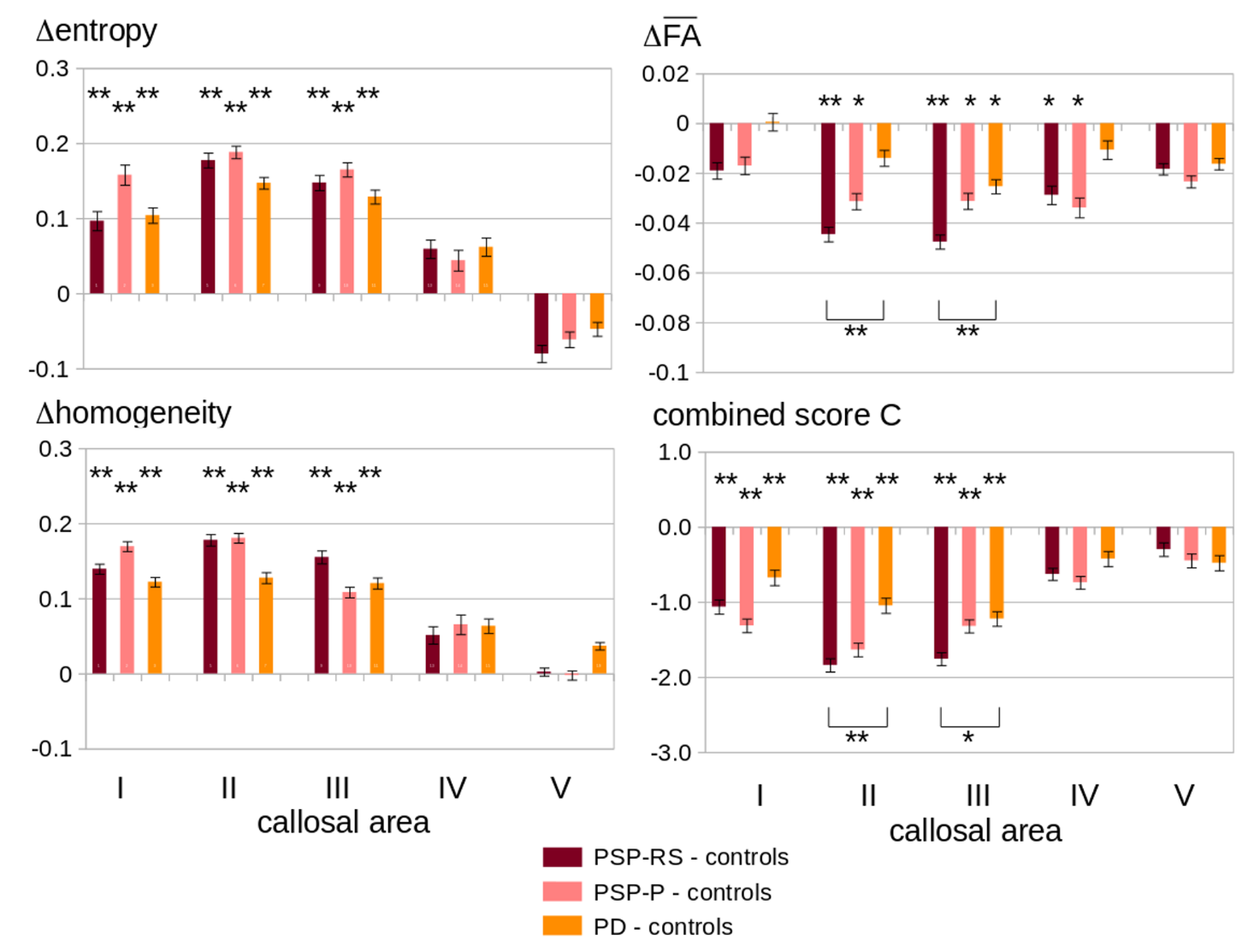


**Supplementary Figure 1.** Subdivision of the PSP group into PSP-RS and PSP-P. **Left panel:** Texture results for CC areas I-V. Displayed are the differences (Δ) in average values of the parameters entropy and homogeneity between the two patient groups and the controls. PSP-RS and PSP-P, as compared to patients with PD and controls. For entropy and homogeneity, no significant differences between PSP-RS and PSP-P were detected. **Right upper panel:** Tractwise fractional anisotropy statistics (TFAS) for fiber tracts originating in callosal areas. Displayed are the differences (Δ) in average FA values between patient groups and controls. PSP-RS show a significant decrease of FA in areas II and III compared to PD, whereas PSP-P showed no significant difference to PD. **Right lower panel:** Combined score C - PSP-RS showed higher alterations than for PSP-P; in areas II and III these alterations (for PSP-RS) were significant compared to PD patients whereas the alterations of PSP-P were similar (not significant) as the alterations for PD patients. Significant differences between PSP subgroups and PD patients were shown by brackets below the column. Error bars are the standard error of the mean. * p < 0.01, ** p < 0.001, corrected for multiple comparisons.
